# The Hahnemannian Consultation Blank

**Published:** 1892-02

**Authors:** 


THE HAHNEMANNIAN CONSULTATION BLANK. Arranged and published by S. Mills Fowler, M. D. Gainesville, Texas, 1891.

This little pamphlet of 5¾ inches by 3 inches, and containing about fifty pages, is designed for physicians as a guide in examining their patients. It contains lists of all the symptoms patients are most likely to have, with spaces for check-marks. The arrangement is very like the lists given in the directions for examination in Dr. Wells' book on Intermittent Fever, now being published in this journal. Dr. Fowler's little book ought to be a great help to the practical physician. W. M. J.

